# Combined 2D and 3D tracking of surgical instruments for minimally invasive and robotic-assisted surgery

**DOI:** 10.1007/s11548-016-1393-4

**Published:** 2016-04-02

**Authors:** Xiaofei Du, Maximilian Allan, Alessio Dore, Sebastien Ourselin, David Hawkes, John D. Kelly, Danail Stoyanov

**Affiliations:** Centre for Medical Image Computing, Department of Computer Science, University College London, London, UK; Centre for Medical Image Computing, Department of Medical Physics, University College London, London, UK; WIREWAX, London, UK; Division of Surgery and Interventional Science, University College London, London, UK

**Keywords:** Instrument tracking and detection, Minimally invasive surgery, Robot-assisted surgery, Surgical vision

## Abstract

**Purpose:**

Computer-assisted interventions for enhanced minimally invasive surgery (MIS) require tracking of the surgical instruments. Instrument tracking is a challenging problem in both conventional and robotic-assisted MIS, but vision-based approaches are a promising solution with minimal hardware integration requirements. However, vision-based methods suffer from drift, and in the case of occlusions, shadows and fast motion, they can be subject to complete tracking failure.

**Methods:**

In this paper, we develop a 2D tracker based on a Generalized Hough Transform using SIFT features which can both handle complex environmental changes and recover from tracking failure. We use this to initialize a 3D tracker at each frame which enables us to recover 3D instrument pose over long sequences and even during occlusions.

**Results:**

We quantitatively validate our method in 2D and 3D with ex vivo data collected from a DVRK controller as well as providing qualitative validation on robotic-assisted in vivo data.

**Conclusions:**

We demonstrate from our extended sequences that our method provides drift-free robust and accurate tracking. Our occlusion-based sequences additionally demonstrate that our method can recover from occlusion-based failure. In both cases, we show an improvement over using 3D tracking alone suggesting that combining 2D and 3D tracking is a promising solution to challenges in surgical instrument tracking.

**Electronic supplementary material:**

The online version of this article (doi:10.1007/s11548-016-1393-4) contains supplementary material, which is available to authorized users.

## Introduction

Detection and tracking of surgical instruments can provide an important information component of computer-assisted surgery (CAS) for MIS [22]. Control systems which can supply automated visual servoing [18], soft motion constraints [19] and tactile feedback [15] are reliant on knowing positional information about both the shaft and the tip of the articulated instrument. Hardware-based solutions such as optical tracking systems using fiducial markers [10] require modification to the instrument design posing ergonomic challenges and additionally suffer from robustness issues due to line-of-sight requirements. Direct use of robotic joint encoders and forward kinematics to track instruments is possible in robot-assisted interventions; however, tendon-driven systems, such as da Vinci$^{\textregistered }$ (Intuitive Surgical Inc., CA), introduce errors in the position information which usually requires correction that can be achieved through visual methods [17, 18]. Entirely image-based solutions [3, 21, 23] directly estimate the instrument pose in the reference frame of the observing camera. This avoids complex calibration routines and can be implemented entirely through software which allows them to be applied retrospectively and without modification to the instruments or the surgical workflow.

Early image-based methods predominantly estimated the instrument pose in 2D by estimating image-based translation parameters, scale and in-plane rotation without explicitly modeling the 3D shape of the instrument. These have been based around low-level image processing [20] which accumulate handcrafted visual features and more complex learned discriminative models [6, 23] which track an instrument by performing detection independently on each frame. Such methods are typically fast and robust, handling complex and fast motion as well as recovery when the instrument is occluded by the field of view of the camera or smoke and tissue as they perform a global or semi-global search of the entire image for the tracked instrument. Fewer methods have attempted to estimate the 3D pose of the instruments directly from image data. This typically is a much more complex problem as it involves estimating three additional degrees of freedom (DOF) from very weak small baseline stereo or monocular cues. However, it provides additional benefits over 2D methods as it allows reasoning about instrument–instrument occlusions and interactions with tissue surfaces. Most of these methods focus on the alignment of a 3D model with a probabilistic classification of the image [1, 2, 16] which allows the fusion of geometric constraints with image data without an offline learning phase. A significant challenge with 3D tracking methods is that they commonly fail when the instrument motion is fast or complex, as they restrict the parameter search to local regions close to the estimated parameters from the previous frame. In many cases, this can lead to drift which requires a manual reset of the tracking.

In this paper, we present a new method which combines the strengths of a novel 2D tracker with a preexisting 3D tracking method [3] allowing us to robustly track surgical instruments through sequences that contain occlusions and challenging motion which cause the 3D tracker to fail. We achieve this by performing global tracking-by-detection in 2D with a keypoint-based tracker which is used to initialize the 3D tracker with image-based translation and rotation parameters as well as an estimate of scale. We then perform a normal gradient-based optimization to estimate the full set of 3D parameters. We quantitatively validate our method using ex vivo data collected from a DVRK controller and forward kinematics and additionally provide convincing qualitative validation on in vivo robot-assisted prostatectomy sequences. Our validation shows the our method provides state-of-the-art 2D tracking performance and significantly improves tracking accuracy in 3D. In the ex vivo sequences, we restrict the motion of rigid 3D tracking as the method we use [3] does not model articulations of the instrument tip. Our validation shows the our method provides state-of-the-art 2D tracking performance and improves tracking accuracy in 3D.

## Methods

**Fig. 1:**
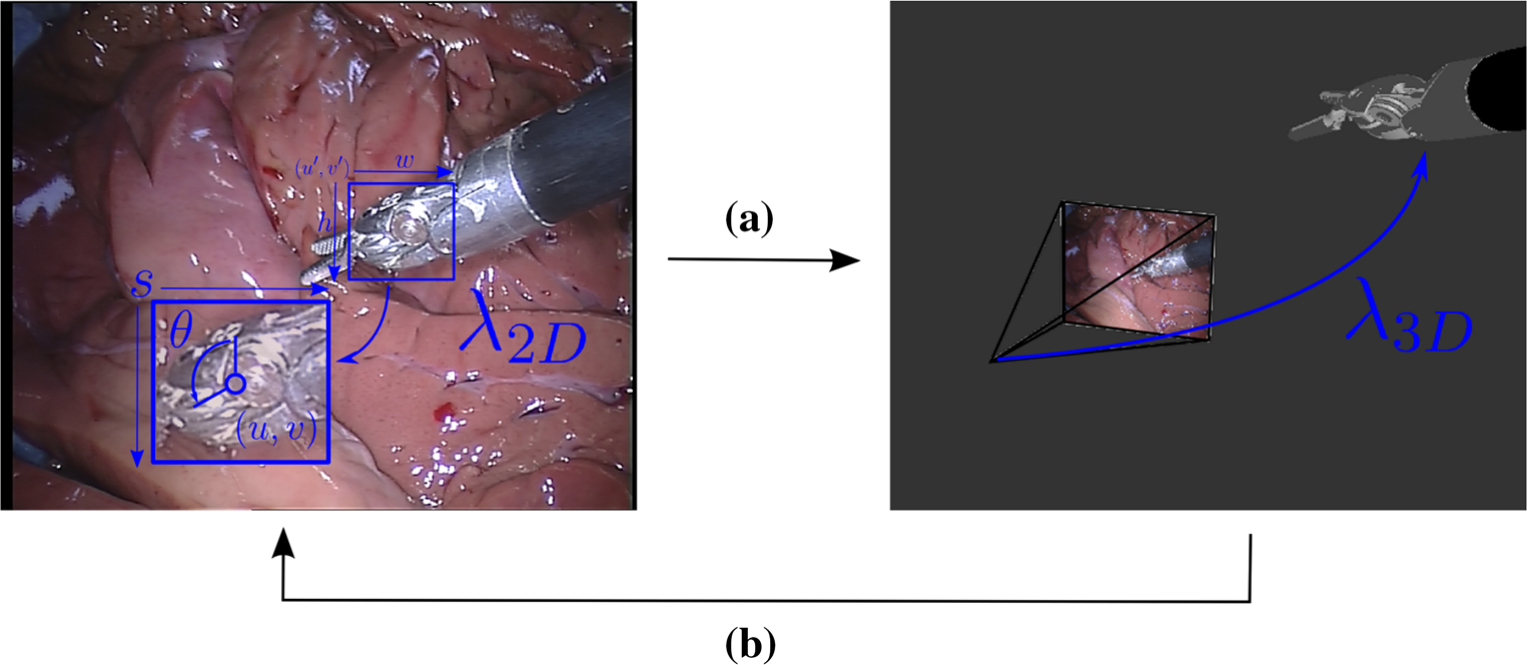
The *left image* shows the 2D detection and estimation of the parameters $\lambda _{2D}$ which are then used **a** to initialize the 3D parameters $\lambda _{3D}$. After the 3D pose is estimated, a new frame is loaded **b** and 2D detection begins again

Our method assumes that we have the 3D pose of the instrument in the first frame which we use to initialize a 2D bounding box $(u^{\prime },v^{\prime },w,h)$ (see Fig. 1) around the instrument head where $(u^{\prime },v^{\prime })$ is the pixel coordinates of top left corner of the bounding box which has width *w* and height *h*. We define the 2D detection problem as the estimation of the parameters $\mathbf {\lambda }_{2D} = (u,v,\theta ,s)$ and the 3D estimation problem as the estimation of the parameters $\mathbf {\lambda }_{3D} = (x,y,z,\phi ,\psi ,\hat{\theta })$, where (*u*, *v*) are the pixel coordinates of the center of the instrument head and $\theta$ is the pitch/in-plane rotation of the instrument shaft around the optical axis. (*x*, *y*, *z*) are the 3D translation coordinate in metric units from the camera coordinate system origin to the instrument coordinate system origin, and $\phi ,\psi ,\hat{\theta }$ are the *x*, *y*, *z* rotations of the instrument in 3D, respectively. For each new input frame, we detect the instrument, estimating the 2D parameters $\mathbf {\lambda }_{2D}$ using our new tracker. Using these parameters, we then initialize a previously developed, open-source 3D tracker [3] which then converges using gradient descent to estimate the full 3D parameter vector $\mathbf {\lambda }_{3D}$.

### Generalized Hough transform for 2D detection

To estimate $\mathbf {\lambda }_{2D}$, we implement a keypoint-based tracker which relies on a Generalized Hough Transform (GHT) [5] and a global histogram segmentation model. The GHT extends the well-known Hough Transform to detect arbitrary shapes as maxima in a parameter space by describing shapes as collections of spatial features in a local coordinate system. Given an example image template containing the object of interest, a reference point which serves as the origin of the local coordinates is computed, usually as the center of the template window. Then, for keypoint-based features (e.g., SIFT [13]) in the template image, the feature orientation and the relative displacement and orientation to the reference point are computed and stored in a database known as an R-table, which fully defines the target object. To perform detection with the GHT, keypoints in a new image are computed and matched to the stored keypoints in the R-table. Each matched keypoint then “votes” for the origin of the coordinate system, and the center is chosen as the reference point with the most votes.

### Initializing the model

Given a sequence of *m* frames $\{I_t\}_{t=1}^{m}$ and the 2D bounding box $(u^{\prime },v^{\prime },w,h)$ on the template frame $I_1$, we detect the parameters $\mathbf {\lambda }_{2D} = (u,v,\theta ,s)$ on every input frame. The object model *M* is represented by a set of keypoints

1 $$\begin{aligned} M=\{(\mathbf {f}_{i,t=1}, d_i, s_{i,t=1})\}_{i=1}^n \end{aligned}$$

where $\mathbf {f}_{i,t=1}$ denotes the *i*th keypoint on the model, $d_i$ represents the distance between keypoint $\mathbf {f}_i$, and the center of the instrument head (*u*, *v*). $s_{i,t}\in \{0,1\}$ is the voting state of keypoint $\mathbf {f}_i$ at frame *t*: 0 for negative and 1 for positive. It is positive if the corresponding keypoint has contributed for the voting of the detected center; otherwise, it is negative. The voting states for all keypoints are initialized as positive for the template frame $I_1$

2 $$\begin{aligned} s_{i,t=1}=1 \quad \forall i\in [1,n] \end{aligned}$$

For each input frame $I_t$ with $t>1$, the keypoints in the model are matched. We gather the matched corresponding keypoints as the vote set $F_{V}$.

3 $$\begin{aligned} F_{V}=\{(\mathbf {f}_{i,t},w_{i,t})\} \quad \forall i\in [1,n] \end{aligned}$$

where $w_{i,t}$ is the voting weight for each matched keypoint $\mathbf {f}_{i,t}$, which is defined based on the segmentation model introduced in Sect. 2.3.

**Fig. 2:**
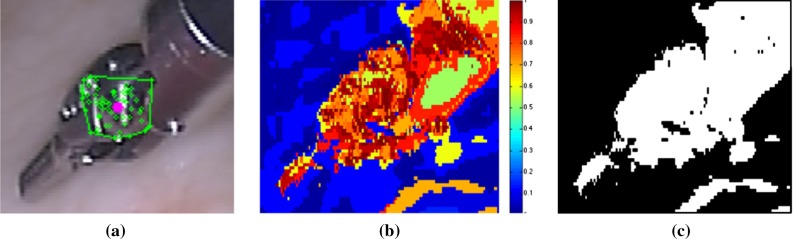
Segmentation model initialization and update strategy: **a** image region inside the convex hull (*green polygon*) of the positive keypoints (*green circle*) is used to initialize and update the foreground histogram; *filled circle* with *magenta color* indicates the reference center; **b** foreground probability colormap illustration, in which *blue color* indicates low probability, while *red color* indicates higher probability; **c** foreground/background classification binary map based on the probability model

### Histogram-based segmentation model

To adapt object model accounting for appearance changes, we are inspired by the work of [7, 8] and we implemented a global probabilistic model based on color histogram by using a recursive Bayesian formulation to better discriminate foreground and background.

The foreground probability of a pixel at frame *t* is based on the segmentation of previous frame $t-1$.

4 $$\begin{aligned} p(c_t=1|y_{1:t})= & {} Z^{-1}\sum _{c_{t-1}}p(y_t|c_t=1)p(c_t=1|c_{t-1})\nonumber \\&\times p(c_{t-1}=1|y_{1:t-1}) \end{aligned}$$

where $c_{t}$ is the class of the pixel at frame *t*: 0 for background and 1 for foreground, $y_{1:t}$ is the pixel’s color from frame 1 to *t*, and *Z* is a normalization constant to keep the probabilities sum to 1. The color distribution $p(y_t|c_t)$ is built with HSV color histograms with $12 \times 12$ bins for H and S channels and 8 separate bins for V channel. We omit the background probability $p(c_t=0|y_{1:t})$ here since it is similar to Eq. 4. The transition probabilities for foreground and background $p(c_t|c_{t-1})$ where $c \in \{0, 1\}$ are empirical choices as in [8], which are not very sensitive.

To detect more boundary keypoints, the bounding box is usually slightly larger than the object, which includes more background pixels. So instead of initializing the histogram from the bounding box on the template image as in [8], we initialize it from the detection result from the first frame after locating the object center. It is assumed that the positive keypoints are most likely located on the object, so we collect all the positive keypoint into $F_{Pos}$

5 $$\begin{aligned} F_\mathrm{Pos} = \{\mathbf {f}_{i,t}\} \quad \text {if } s_{i,t}=1 \quad \forall \mathbf {f}_{i,t}\in F_{V} \end{aligned}$$

The foreground histogram is then initialized from the image region inside the convex hull of all the positive keypoints $CH(F_\mathrm{Pos})$, which contains less background pixels. The background histogram is initialized from the image region surrounding the detected object bounding box (with some margin between). For the following frames, the color distributions are adapted in the same way as the initialization (shown in Fig. 2)

6 $$\begin{aligned} p(y_t|c_t=1)= & {} \delta p(y|y\in CH(F_\mathrm{Pos}))\nonumber \\&+(1-\delta )p(y_{t-1}|c_{t-1}=1) \end{aligned}$$

where $\delta =0.1$ is the model update factor.

The voting weight of a keypoint is defined as the mean foreground probability of the image patch surrounding the keypoint

7 $$\begin{aligned} w_{i,t} = p(c_t=1|\mathbf {f}_{i,t}) \end{aligned}$$

During the voting process, we set the weight threshold $w_\mathrm{thres}=0.5$, only keypoints with higher weight $(w_{i,t}>w_\mathrm{thres})$ participate in the voting process, and the weighted votes accumulated based on the segmentation model. In regard to the voting, we developed a rotation-invariant voting scheme in Sect. 2.4.

### Rotation-invariant Hough voting scheme

**Fig. 3:**
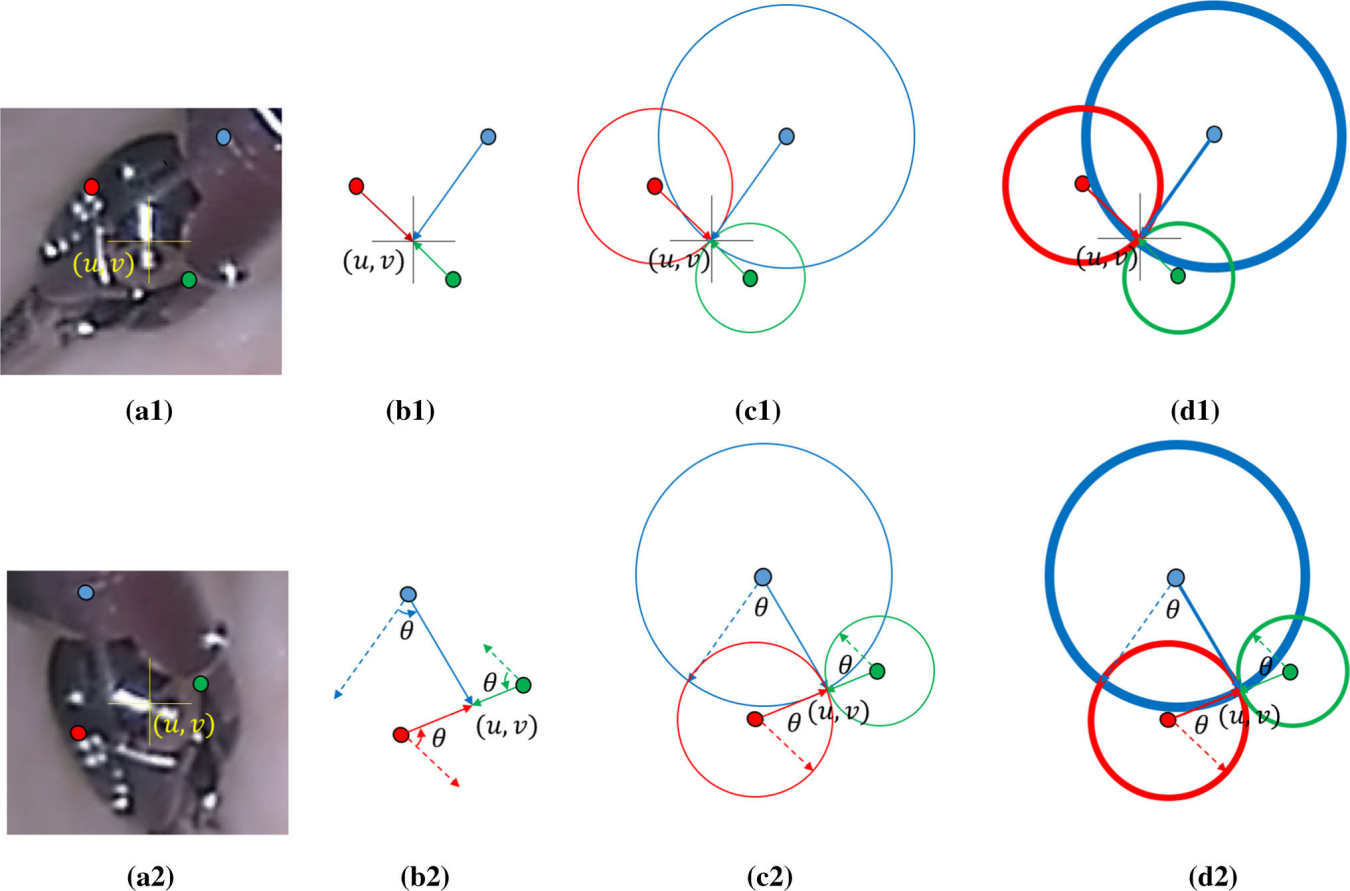
Voting scheme illustration: **a1** keypoints and reference center on the model (shown in *color*); **a2** keypoints and the tracked center (*u*, *v*) on the input frame; in [14], keypoints vote for the reference center (**b1**); in the input frame, the rotation $\theta$ is estimated by pairwise angular change and vote based on the rotation estimation in (**b2**); our rotation-invariant voting scheme votes not only for one direction, but a *circle* (**c1**, **c2**), in order to improve robustness, keypoint votes for a *ring circle*, and the rotation $\theta$ and scale *s* are estimated after voting (**d1**, **d2**)

When the object undergoes scale change or in-plane rotation, the voting also needs to rotate and scale in order to locate the object center. Scale and rotation information can be obtained from most feature detectors, but since it is usually not reliable enough, in [14], the authors analyzed the pairwise Euclidean distance and angular change between keypoints. We illustrated their voting scheme and ours in Fig. 3: Keypoints on the model and on the input frame are matched in Fig. 3a1, a2; then in the input frame, median pairwise angular change between keypoints is computed by comparing with the initial constellation in Fig. 3b1, and correspondent keypoints rotate votes based on the median angular change $\theta '$ in Fig. 3b2. It displays the ideal situation for rotation estimation, but when the percentage of outliers is high, votes will probably miss shoot the center based on unreliable rotation estimation. We develop a rotation-invariant voting strategy shown in Fig. 3c1, c2. For each keypoint, instead of voting for only one direction, it votes for a circle. In this way, our vote scheme does not rely on any pre-estimation of rotation, and the maximum vote still accumulated at the center without any potential error induced by the pre-voting rotation estimation. In order to improve the overshooting or fall short situation for scale estimation or out-of-plane rotation, we make it more robust by voting for a ring circle in Fig. 3d1, d2. The thickness ratio $r_d$ is set to be [0.95, 1.05]. The initial scale $s_{t=1}$ is set to be 1.0, the radius of the voting circle $d_{i,t}$ is based on the scale of the previous frame $s_{t-1}$, and the distance of the keypoint to the reference center of the model $d_i$ is

8 $$\begin{aligned} d_{i,t} = r_d*d_i*s_{t-1} \end{aligned}$$

After voting, the scale $s_t$ and rotation $\theta _t$ are estimated based on the scale change and angle change of all the positive keypoints.

### Model adaptation

One of the challenges for visual tracking is how and when to adapt the object model to cope with appearance changes due to deformation, illumination variations, etc. In endoscopic images, when the object center is out of view or out of plane, instead of updating the model, we have to reset the detector to re-detect the object. To achieve this, we define the following updating strategy. Whenever the voted center is out of the convex hull of the positive keypoint set $F_\mathrm{Pos}$, we evaluate all the keypoints inside the bounding box $B_{t}$ around the detected center based on the segmentation model. If the weight $w_t^C$ of the keypoint candidate $\mathbf {f}_t^C$ is higher than the weight threshold $w_\mathrm{thres}$, it is considered as a potential keypoint and is included in the keypoint candidate set $F_\mathrm{candi}$; otherwise, it will be discarded.

9 $$\begin{aligned} F_\mathrm{candi}=\{\mathbf {f}_{t}^C\}\quad \text {if } w_t^C>w_\mathrm{thres} \quad \forall \mathbf {f}_{t}^C\in B_{t} \end{aligned}$$

Then, we analyze the distribution of the keypoint candidates with regard to the object center: (i) If the center (*u*, *v*) is inside the convex hull of the candidates $F_\mathrm{candi}$ and the number of candidates is higher than certain threshold, we add the new candidates $M_\mathrm{candi}$ into the model and remove negative features, and then use the updated model to continue tracking; (ii) if the center is outside of the convex hull, it indicates the object is most likely out of image or is under out-of-plane rotation, so we switch the detector into reset mode: If the object is matched, the detector will be switched back to normal mode.

**Fig. 4:**
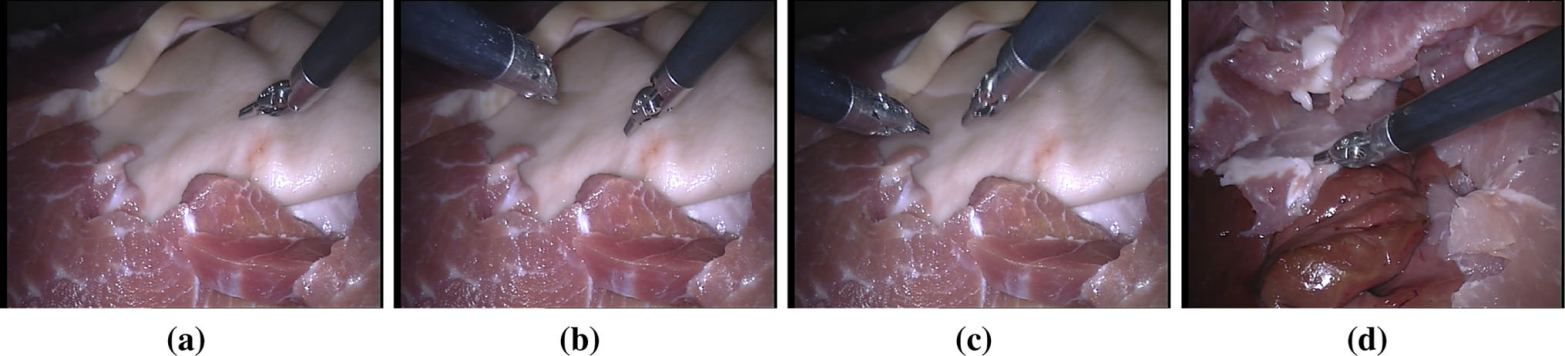
Example frames from our ex vivo sequences acquired using a da Vinci$^{\textregistered }$ (Intuitive Surgical Inc., CA) classic stereo laparoscope. The images show typical challenges in instrument tracking, such as instrument and tissue-based occlusions and sequences where the instrument goes in and out of view repeatedly

**Table 1:** Numerical results for the 3D tracking for each of the ex-vivo sequences

	Dataset I	Dataset II	Dataset III	Dataset IV
2D3D	3.70 $\pm$ 2.28	16.23 $\pm$ 11.83	8.29 $\pm$ 11.29	11.54 $\pm$ 7.94
3D only	4.76 $\pm$ 3.28	38.47 $\pm$ 32.11	51.37 $\pm$ 52.10	16.79 $\pm$ 14.88

Each value shows the mean error (mm) of the translation error for our 2D3D method and for the 3D only tracking

### Combining 2D and 3D tracking

We use an open-source 3D level set tracker [3] which is capable of recovering the full 3D pose of surgical instruments by aligning multiple-level set segmentations with Random Forest pixel classifications and additionally uses optical flow tracking to local track features on the instrument body. We use the 2D pose $\mathbf {\lambda }_{2D}$ to initialize the 3D pose of this method in each frame, rather than using the tracking-by-initialization method of the original authors. The parameters (*x*, *y*, *z*) of $\mathbf {\lambda }_{3D}$ are initialized by ray casting (*u*, *v*) and using the *z* estimate from the first frame, scaled by *s*. $\theta$ is used directly to initialize $\hat{\theta }$, and $\phi ,\psi$ are retained from the previous frame. Effectively, we only retain the parameters in the 3D tracker which cannot be estimated by the 2D tracker. Given an initial estimate, we allow the 3D level set-based tracker to converge to a solution through gradient descent.

## Experiments and results

In this section, we present validation on both our novel 2D tracker “GHT” and our 2D-initialised-3D (referred to as 2D3D) tracking. In this section, we refer to the 3D tracker without 2D initialization [3] as “3D only.” Our quantitative validation is performed on new ex vivo datasets which we have made available online (see Fig. 4).^1^ We hope that by releasing data, we will encourage other researchers to test their methods against our data, an idea which was explored recently in the Endoscopic Vision Challenge at MICCAI 2015 which provided labeled segmentation and tracking data for laparoscopic and robotic-assisted minimally invasive surgery.

### Ex vivo experiments

To evaluate the ability of our method to robustly track a surgical instrument through challenging sequences, we constructed four datasets with porcine tissue samples. Our *ex vivo* sequences are collected using a da Vinci$^{\textregistered }$ (Intuitive Surgical Inc., CA) robot where we obtained joint encoder data from a DVRK controller box [12]. Using forward kinematics, we can compute the 3D transform for the instrument in the reference frame of the stereo camera using manual calibration to remove the offset between the robot and camera coordinate system. This can be projected into the image plane to obtain validation for both the 2D and 2D3D tracking. We compare our 2D tracking method with the-state-of-art CST tracker [9] and TLD tracker [11] using precision and box plots based on location error metric and area under curve (AUC) to analyze the performance. These metrics are widely used to evaluate tracking performance [4, 24]. Precision plots show the percentage of frames (*y* axis) where the estimated position (*u*, *v*) is within a distance threshold (*x* axis) compared with the ground truth. In the box plot, edges of the box are 25 and 75 % percentiles, the whiskers extend to the most extreme data points not considered outliers, and the red markers are outliers plotted outside the box.

**Fig. 5:**
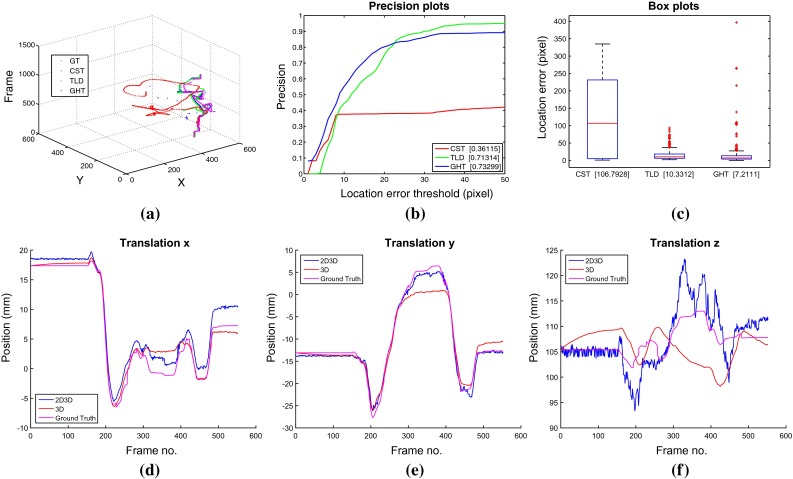
Performance comparison for dataset I, which contains a tool occlusion between frames 250–400

We compare our 2D tracking method with the-state-of-art CST tracker [9] and TLD tracker [11] using precision and box plots based on location error metric and area under curve (AUC) to analyze the performance. These metrics are widely used to evaluate tracking performance [4, 24] and show the percentage of frames where the estimated position is within a threshold of the ground truth. We also summarise the numerical results for the 3D tracking in Table 1. In the table, mean translation errors for our 2D3D method and the 3D only tracking are shown for each of the ex vivo sequences.

#### Tracking through occlusions and out of view

**Fig. 6:**
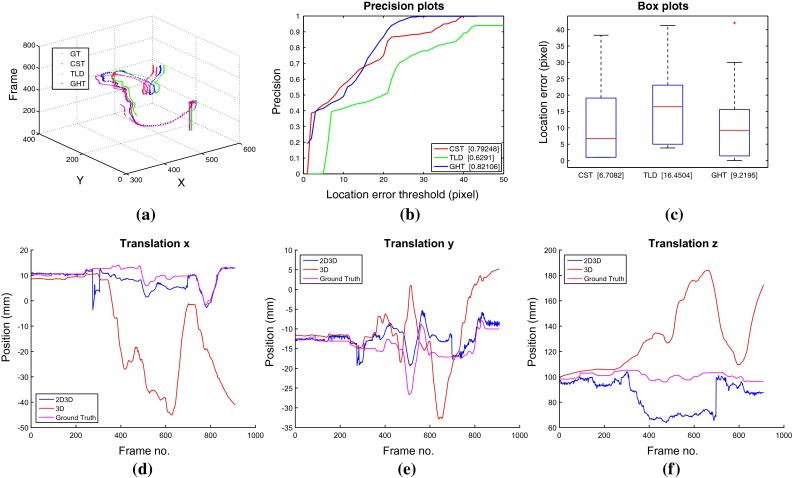
Performance comparison for dataset II, which contains a tissue occlusion between frames 225–350

**Fig. 7:**
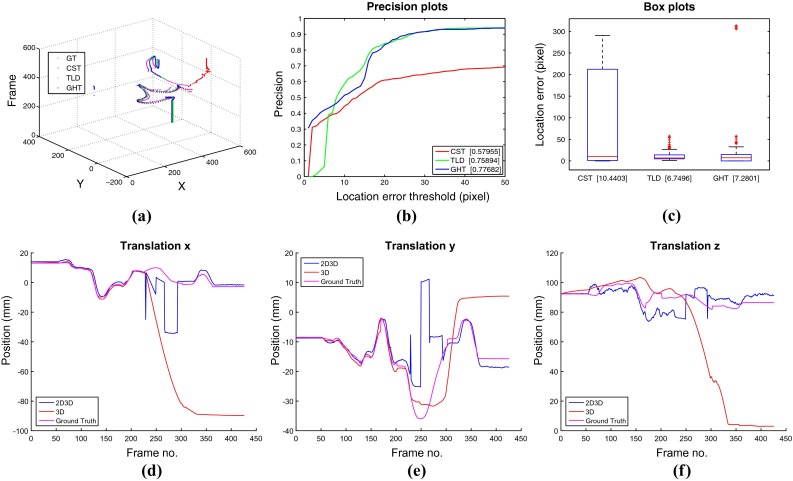
Performance comparison for dataset III, which contains out-of-view occlusions between frames 325–350

Dataset I evaluates the ability of the method to track instruments when they are occluded by other instruments, effectively assessing our method’s ability to avoid tracking association errors between the target instrument and additional instruments in the frame, even when they violate each other’s image space. Dataset II evaluated the ability of the method to track instruments when they are occluded by tissue samples. Dataset III and dataset IV evaluate the ability of our method to recover when the instrument moves out of view of the camera. The trajectories of the tracked center and the precision plots for each sequence are shown in Figs. 5 and 6. The CST tracker lacks the ability to recover from occlusions or out-of view situations compared with the TLD tracker and our GHT tracker. Our GHT tracker has the highest AUC score among three trackers, which means our method can handle various occlusion and out-of-view challenges. The 3D tracker demonstrates similar performance with and without 2D initialization in Dataset I, with slight improvement in the *z* axis estimation during the occluded frames. Dataset II clearly demonstrates the improvement of our method as the 3D only tracker loses tracking at frame 380 and never recovers. The same effect occurs in dataset III where the 3D only tracker loses tracking after occlusion and does not recover. Figures  5, 6 and 7a show the trajectories of the tracked center for three 2D methods, (b) show the precision plot for three 2D methods, (c) show the box plot for three 2D methods, and (d–f) show the 3D trajectory of the proposed 2D3D tracker compared with using the 3D tracker directly. Dataset I evaluated the ability of the method to track instruments when they are occluded by tissue samples. Dataset II evaluates the ability of the method to track instruments when they are occluded by other instruments, effectively assessing our method’s ability to avoid tracking association errors between the target instrument and additional instruments in the frame, even when they violate each other’s image space. Dataset III evaluates the ability of our method to recover when the instrument moves out of view of the camera. The trajectories of the tracked center and the precision plots for each sequence are shown in Figs. 5, 6 and 7. The CST tracker lacks the ability to recover from occlusions or out-of view situations compared with the TLD tracker and our GHT tracker. Our GHT tracker has the highest AUC score among three trackers, which means our method can handle various occlusion and out-of-view challenges. The 3D tracker demonstrates similar performance with and without 2D initialization in Dataset I, with slight improvement in the *z* axis estimation during the occluded frames. In Dataset II, however, the improvement is significant as the 3D only tracker loses tracking at frame 380 and never recovers. The same effect occurs in dataset III where the 3D only tracker loses tracking after occlusion and does not recover. Figures  5, 6, 7 (a) show the trajectories of the tracked center for three 2D methods, (b) show the precision plot for three 2D methods, (c) show the box plot for three 2D methods, and (d–f) show the 3D trajectory of the proposed 2D3D tracker compared with using the 3D tracker directly.

**Fig. 8:**
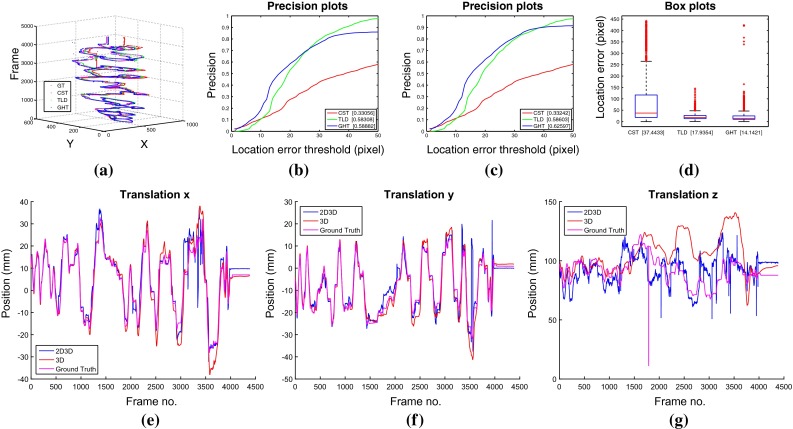
Performance comparison for the extended tracking sequence, dataset IV

**Fig. 9:**
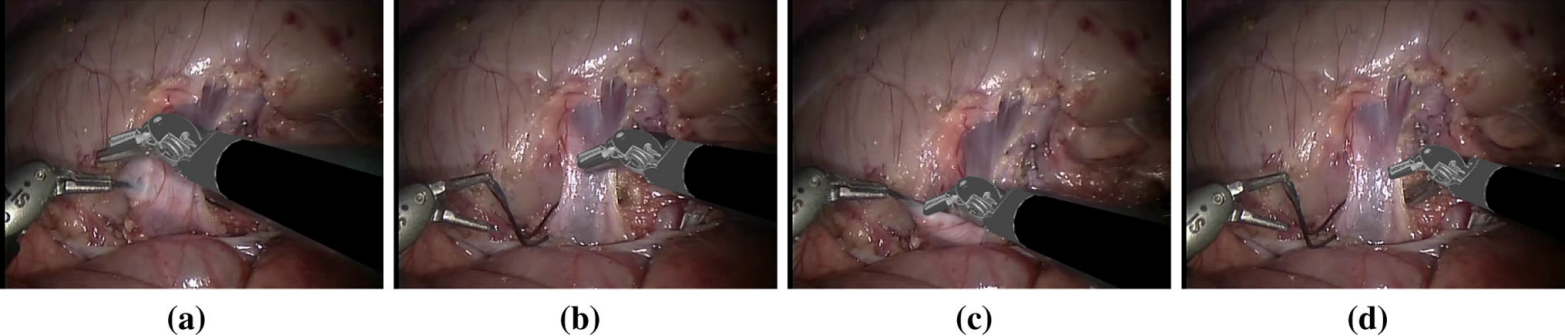
Frames showing an instrument tracked through an in vivo sequence. **a**–**c** Demonstrate good accuracy, whereas in **d** a failure mode for our algorithm is exhibited where poor classification on the instrument body causes the 3D tracked to fail to converge correctly

#### Long-term tracking

We construct an extended sequence (dataset IV) of over 4000 frames to demonstrate the capability of our method to track the pose of the instrument in 3D without failing from drift. We display the results in Fig. 8 where (a) shows the trajectories of tracked center, (b) shows a precision plot for three 2D methods over the whole sequence, (c) shows a precision plot for three 2D methods over frames where all methods report a positive detection, (d) shows a box plot for the three 2D methods, (e–g) show the 3D trajectory of the proposed 2D3D tracker compared with using the 3D tracker directly. The figures show that our method is capable of reliable long-term tracking although it does exhibit interesting failure cases. On dataset IV, our method fails to discriminate between out-of-view occlusions and out-of-plane-based appearance changes which results in non-detected output when the tracked points are rotated out of the field of view or such that the appearance of the patch changes beyond recognition. To display quantitative precision plot results in cases where each 2D detection method has some false non-detections, we display 2 types of plot: one where we display the results from the whole sequence where we set an infinite distance for missed detections (Fig. 8b) and one where we only consider frames where all of the 2D tracking methods report a detection (Fig. 8c).

### In vivo experiments

We additionally qualitatively validate our method using robotic video data [16]. Example images showing our method performing detection on these images are shown in Fig. 9. This in vivo sequence shows that our method is capable of tracking through complex surgical images even when the instrument undergoes articulation, which our method does not explicitly model.

## Conclusions

In this paper, we present a novel GHT-based 2D tracker with a global histogram probabilistic segmentation model which we combine with a 3D tracking algorithm to robustly estimate the full 3D pose of instruments in minimally invasive surgery. Our extensive ex vivo validation demonstrates that our method is not only capable of tracking instruments over extended sequences but that it can also recover from tracking failures and occlusions, a feature that has not been demonstrated in any prior 3D tracking work in a minimally invasive surgical context. Future improvements to this method will focus around removing the requirement on a manual initialization. This can potentially be achieved with an enforced fixed position of the instrument, while the 3D pose estimator converges to a correct solution.

## Electronic supplementary material

Below is the link to the electronic supplementary material.

Supplementary material 1 (mp4 36653 KB)

## Footnotes

1 www.surgicalvision.cs.ucl.ac.uk/benchmarking.
